# How and Why Context Matters: A Personal Reflection: Comment on "Stakeholder Perspectives of Attributes and Features of Context Relevant to Knowledge Translation in Health Settings: A Multi-Country Analysis"

**DOI:** 10.34172/ijhpm.2022.6782

**Published:** 2022-04-27

**Authors:** Alison Kitson

**Affiliations:** College of Nursing and Health Sciences, Caring Futures Institute, Flinders University, Adelaide, SA, Australia

**Keywords:** Context, PARIHS, Implementation Success

## Abstract

This commentary acknowledges that the evidence-based practice (EBP) movement did not automatically or initially understand the impact of context on successful implementation (SI). The subsequent work of research teams, such as the PARIHS (Promoting Action on Research in Health Services) team, and the Ottawa team led by Squires, have contributed to the ongoing refinement of the concept. However, still under discussion is whether having a more comprehensive set of contextual attributes will necessarily lead to more implementation success. Just as the strength of the evidence does not automatically lead to implementation success, so having a comprehensive understanding of contextual factors will not necessarily improve implementation uptake.

 It is now rather surprising to consider that when the evidence-based practice (EBP) movement hit the world stage in the early 1990s, context was not even considered to be an important variable in the quest to get evidence into practice. Dominating the discourse was the pursuit of research excellence, as demonstrated by the adherence to rigorous systematic review training and level of evidence. As a young researcher working in Oxford in the 1990s, I had the privilege of being at the epicentre of this movement, motivated by leaders such as Iain Chalmers, Muir Gray and Dave Sackett. Theirs was the voice of pure epidemiological reason and they generated a way of thinking about EBP that has arguably changed healthcare practice.

 My job at that time was to lead the setting up of a clinical practice research unit for nursing. This entity, called the National Institute for Nursing, was co-funded by the Royal College of Nursing of the United Kingdom, Oxford District and Regional Health Authorities and a philanthropic organisation called the Sainsbury’s Monument Trust. The Institute was also supported by Oxford University via Green (Templeton) College and Oxford Brooks University Nursing Department. Our job was to start the journey of generating the evidence base for clinical nursing practice and to get it into practice. Interesting to note also was the attention paid to aligning key organisations to a new initiative in order to optimise the success of the venture. This was probably my first lesson in understanding the importance of (the political and organisational) context at a macro level.

 The PARIHS framework^1^ (Promoting Action on Research in Health Services) was developed in response to the challenges we were experiencing in our attempts to get clinical guidelines and other pieces of evidence into practice. The pure logic of the EBP movement just was not working for us. So, this was when we generated the formula that successful implementation (SI) was a function (f) of the interplay of the type of* evidence* (E), the *context* (C), and the way the evidence was helped, enabled or *facilitated *to get into practice (F). The PARIHS framework was one of the first implementation science or knowledge translation frameworks to make this bold statement that factors other than the quality of evidence could influence effective uptake. It must also be noted that in other disciplines (eg, organisational theory and psychology), context was a well-recognised construct known to shape processes and outcomes.

 As Squires et al^2^ have indicated the early PARIHS framework identified three key elements to context, namely *leadership*, *culture* and *feedback*. These dimensions were not conceived as being inclusive of all contextual factors, but reflective of the priority elements that were generated inductively from an analysis of four case studies that helped to test the early PARIHS framework. Concept analyses of the three core elements^3-5^ of PARIHS followed (McCormack et al on context; Harvey et al on facilitation; Rycroft-Malone et al on evidence). As a result of the context concept analysis, we further refined the framework to include *resources* as a core context element and expanded audit and feedback to the broader concept of *evaluation*. These refinements were presented as part of our work on developing and refining ways to operationalise the framework.^6^

 A further refinement of the framework took place in 2015^7^ when, in response to feedback from users, we refined the original PARIHS framework and called it the integrated or i-PARIHS framework. *Facilitation* became the active ingredient moderating the other key elements: evidence was extended to embrace wider notions of new ideas and was termed *innovation; *we took account of the fact that the individuals or teams that were going to use the new evidence were invisible in the original framework, hence the addition of a construct called ‘*recipients.*’ But the biggest refinement was to our representation of *context. *From a linear representation of items located at (implicitly) at unit or ward level, we argued that *context* was a multi-level (macro, meso, micro) and multidimensional construct. The facilitator’s (or whomever was enabling the process of the uptake of new knowledge) job was to work with the recipients and stakeholders to diagnose what was necessary to do in relation to the innovation, recipients and the *inner (local), inner (organisational) and outer contexts.* Such diagnostic activity is common with other frameworks, some concentrating on behavioural characteristics of individuals^8^; others providing data about contextual or organisational readiness for change data.^9^

 The primary question regarding Squires and colleagues’^2^ painstaking work on identifying characteristics of context is whether having a comprehensive list will in fact lead to improvements in facilitators’ ability to improve the uptake of evidence into practice. Implicit in their argument is that continued under-achievement of successful evidence implementation is related to this failure to describe and hence measure, modify, and control context. I am not convinced that there is such a clear correlation. Indeed, the crux of Squires and colleagues’^2^ argument seems to rest on the premise that if contextual factors are thoroughly described and accounted for, that implementation will become easier or more successful. This line of argument is reminiscent of the early EBP days when it was assumed that the strength of the evidence would be an advantage to SI. We now know this is not the case, hence the move towards multidimensional approaches to understanding the science of implementation. Having a list and providing a context assessment to a team of clinicians who have not had any experience in implementation could be a daunting experience, so the facilitator or knowledge broker needs to actively engage with stakeholders in the immediate context to help them work out what to prioritise as they go forward with their change activity. Implementation is a profoundly social enterprise.

 In our research on developing a set of tools to help clinicians and researchers use the i-PARIHS framework,^10,11^ we have constantly had to work out the balance between providing a comprehensive view of the constructs (*innovation, recipients, inner (local) context, inner (organisational) context and outer context*) and keeping the tools sufficiently clear, short and simple so that everyone gets a sense of the key issues. Also, given the inherent worldview of our approach to knowledge translation^10^ and implementation which is that it is multidimensional, contingent, and more characteristic of a complex adaptive system, the challenge then becomes one of not being prescriptive but rather the tools are used to inform, shed light and help tailor multiple small actions to work towards the goal of implementation.

 Squires et al^2^ have moved the conversation on in terms of outlining a comprehensive list of contextual factors that could influence SI. How they are operationalised and whether they have increased impact because of their specificity across health areas are still to be tested.

## Ethical issues

 Not applicable.

## Competing interests

 AK is one of the originators of the PARIHS framework.

## Author’s contribution

 AK is the single author of the paper.
